# Supplementary material for: Complex‐centric proteome profiling by SEC‐SWATH‐MS
Source: Mol Syst Biol. 2019 Jan 14;15(1):e8438. doi: 10.15252/msb.20188438 (PMC6346213; doi:10.15252/msb.20188438)
Supplement: Supplementary file 8 — Dataset EV7 [file MSB-15-e8438-s008.zip › feature_plots_string/P48735.pdf]

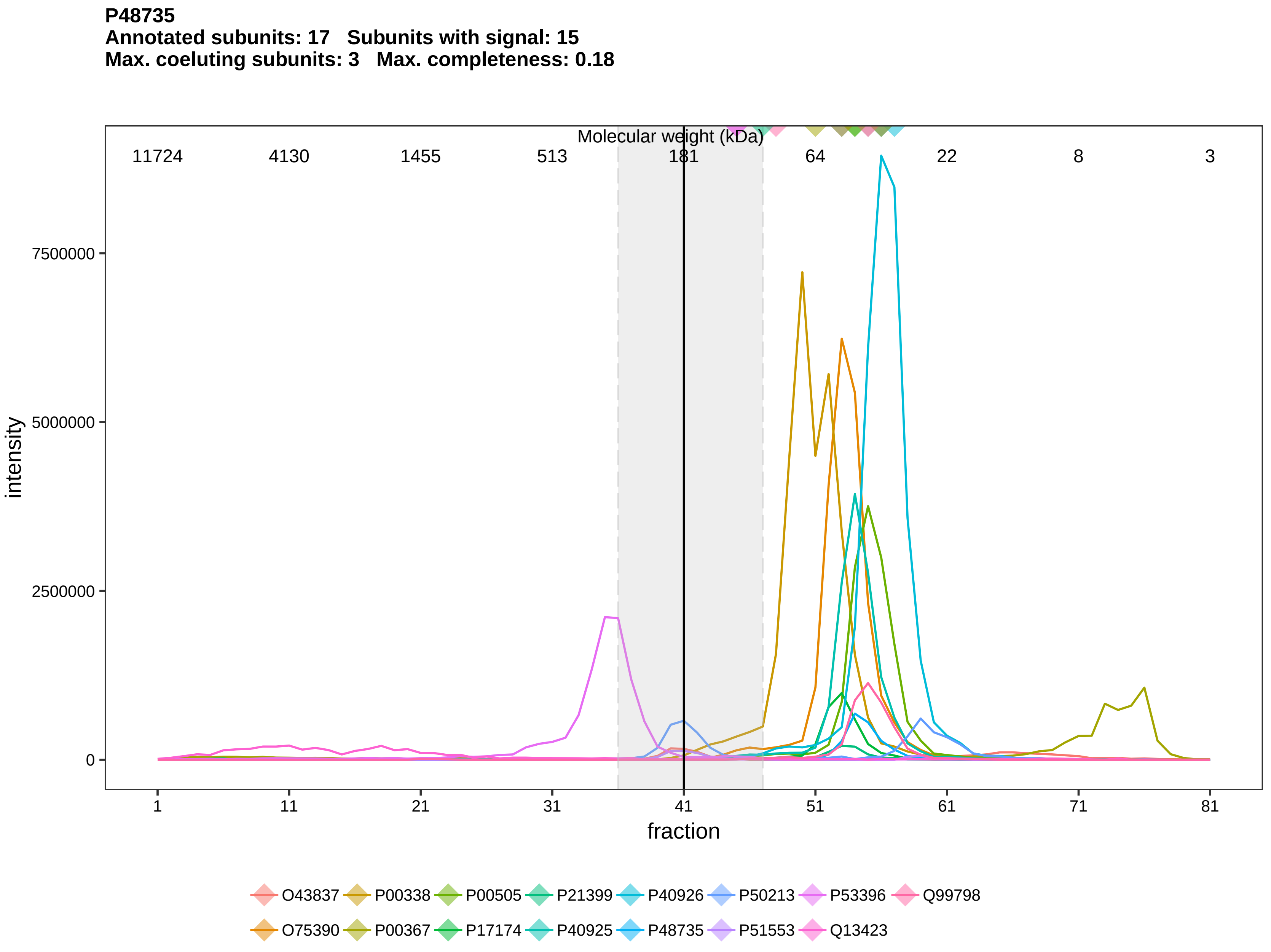

P48735
Annotated subunits: 17 Subunits with signal: 15
Max. coeluting subunits: 3 Max. completeness: 0.18
Molecular weight (kDa)
11724
4130
1455
513
181
64
22
8
3
intensity
7500000
5000000
2500000
0
1
11
21
31
41
51
61
71
81
fraction
O43837
P00338
P00505
P21399
P40926
P50213
P53396
Q99798
O75390
P00367
P17174
P40925
P48735
P51553
Q13423
